# Supplementary material for: Different selection practices affect the environmental sensitivity of beef cattle
Source: PLoS One. 2021 Apr 2;16(4):e0248186. doi: 10.1371/journal.pone.0248186 (PMC8018670; doi:10.1371/journal.pone.0248186)
Supplement: S1 Table — (DOCX) [file pone.0248186.s001.docx]

**S1 Table.** Covariance component for select weight (SW), scrotal circumference (SC) and days to first calving (DFC) for each selection line given by reaction norm model.

| **Component** |  | **Mean** | **SD** | **PPI95%** | |
| --- | --- | --- | --- | --- | --- |
| **Variance of intercept** | | | | | |
| SW | NeC | 808.16 | 140.10 | 533.55 | 1,082.80 |
|  | NeS | 1061.00 | 82.36 | 899.55 | 1,222.40 |
|  | NeT | 1342.80 | 89.70 | 1,166.90 | 1,518.60 |
| SC | NeC | 4.07 | 1.17 | 1.77 | 6.37 |
|  | NeS | 4.19 | 0.77 | 2.69 | 5.70 |
|  | NeT | 6.33 | 0.76 | 4.84 | 7.82 |
| FDC | NeC | 645.52 | 332.88 | 12.67 | 1,278.40 |
|  | NeS | 221.84 | 121.31 | -15,93 | 459.61 |
|  | NeT | 356.60 | 175.47 | 12.673 | 700.52 |
| **Variance of slope** | | | | | |
| SW | NeC | 105.33 | 43.21 | 20.64 | 190.03 |
|  | NeS | 47.76 | 22.39 | 3.87 | 91.65 |
|  | NeT | 262.67 | 52.91 | 158.96 | 366.38 |
| SC | NeC | 0.76 | 0.41 | -0,05 | 1.56 |
|  | NeS | 0.27 | 0.19 | -0,11 | 0,65 |
|  | NeT | 0.96 | 0.35 | 0.28 | 1.65 |
| FDC | NeC | 282.88 | 194.31 | -97,97 | 663.74 |
|  | NeS | 79.60 | 54.92 | -28,04 | 187.24 |
|  | NeT | 140.35 | 69.18 | 4.76 | 275.94 |
| **Covariance between intercept and slope** | | | | | |
| SW | NeC | 209.00 | 38.26 | 134.01 | 284.00 |
|  | NeS | 172.70 | 29.48 | 114.92 | 230.49 |
|  | NeT | 18.54 | 38.14 | -56,21 | 93.28 |
| SC | NeC | 0.05 | 0.31 | -0,56 | 0.66 |
|  | NeS | 0.33 | 0.26 | -0,18 | 0,84 |
|  | NeT | -0,35 | 0,28 | -0,90 | 0,20 |
| FDC | NeC | -179,9 | 170.64 | -514,35 | 154.55 |
|  | NeS | -50,88 | 67.31 | -182,81 | 81,05 |
|  | NeT | -188,79 | 84.44 | -354,29 | -23,29 |
| **Residual variance** | | | | | |
| SW | NeC | 296.02 | 37.71 | 222.10 | 369.94 |
|  | NeS | 505.33 | 25.21 | 455.92 | 554.74 |
|  | NeT | 582.79 | 30.41 | 523.18 | 642.39 |
| SC | NeC | 1.60 | 0.39 | 0.83 | 2.36 |
|  | NeS | 2.18 | 0.27 | 1.65 | 2.71 |
|  | NeT | 2.41 | 0.26 | 1.89 | 2.92 |
| FDC | NeC | 731.51 | 125.88 | 484.78 | 978.25 |
|  | NeS | 841.21 | 64.37 | 715.06 | 967.39 |
|  | NeT | 841.40 | 79.85 | 684.89 | 997.91 |

SD=standard deviation; PPI=posterior probability interval. NeC, control; NeS, selection; and NeT, traditional
