# Supplementary material for: Different selection practices affect the environmental sensitivity of beef cattle
Source: PLoS One. 2021 Apr 2;16(4):e0248186. doi: 10.1371/journal.pone.0248186 (PMC8018670; doi:10.1371/journal.pone.0248186)
Supplement: Attachment — Submitted filename: Response to Reviewers.docx [file pone.0248186.s002.docx]

**Reviewers' comments:**

**Reviewer's Responses to Questions**

**Reviewer #1:** I compliment the authors on this work. It is a very good data set and an interesting project. I don't have concerns about the analysis or presentation of results. I do have some concern about possible over-interpretation of results. Some findings are certainly as expected, less GxE for SW than DFC, for example. We have found similar trends for growth traits versus stayability. I am not convinced that differences among the selection lines should be strong enough to explain the differences among the Spearman correlations presented in Table 2. These correlations vary quite markedly. How reliable are they? What are the biological explanations for these differences. Further, it would enhance the value of the paper to have greater discussion of the implications of the work. Even a few sentences would be helpful. **The paragraph about Spearman correlation has been rewritten for better understanding (L375-384).** **We added two paragraphs to the discussion as suggested (L505-507 and 518-522).**

**Reviewer #2:** This manuscript addresses an important topic, is scientifically sound and well written. Below are some suggestions and a couple of errors that need to be corrected.

108 Should it read “Starting in 2012…” **It was corrected as indicated (L106).**

109 More detail needs to be given on this selection scheme and how this altered the selection for weight. **A reference was added to give details of NeT line. The inclusion of residual feed intake in the selection criterium of NeT line should not affect the weight selection since the genetic correlation between SW and RFI is low (Ceacero et al., 2016-PLosOne). However, the genetic gain in SW is expected to diminish since the animals have been selected for two traits after 2012 (L106-108).**

**- CEACERO TM, MERCADANTE MEZ, CYRILLO JNSG, CANESIN RC, BONILHA SFM, ALBUQUERQUE LG, HANSEN PJ. Phenotypic and Genetic Correlations of Feed Efficiency Traits with Growth and Carcass Traits in Nellore Cattle Selected for Postweaning Weight. Plos One, v. 11, p. e0161366, 2016.**

120 This should read: …by adding the adjusted weight…” **It was corrected as indicated (L119).**

123 Same as above, add adjusted. **It was corrected as indicated (L122).**

126 This is confusing, "what is meant by SW of females was adjusted later"? Wasn't this already explained in the preceding sentences? If so, this sentence should be eliminated. **Phrase eliminated as indicated.**

131 Should this be "outside the expected calving season"? If so, how was that determined? **Sorry, it was a mistake. The sentence was corrected according to the data edition (L129-130).**

147 Should it be “…had no records for some studied years.”? **It was corrected as indicated (L146).**

177 How would selection for weight impact this covariate? **In the preliminary analysis of variance, it was found that the weight at entry into the breeding season was significant for DFC, since even though the heifers' age difference was significant, it was only 3 months.**

229 Shouldn't these be reversed? Unfavorable CG should have the greatest number of days to first calving. **You are right. It was corrected as indicated (L228).**

252 Based on the Figure 1, I think these are the values for DFC and the values listed for DFC are for SC. Either the text is wrong or the figure is wrong. **The text was wrong, but it has already been corrected (L250-251 and L254-255).**

258 A table with these correlations may be helpful. **We agree that the two forms of presentation are interesting. If this is not a requirement, we prefer to present the correlations in the text. OK?**

369 For the other two traits you reported the percentage of plastic and extremely plastic; I recommend being consistent in reporting. **It was changed as recommended (L367-369).**

407 This was not a response to selection, there was no selection for SC. We would expect a satisfactory response to selection for SC based on the heritabilities. **It was changed as recommended (L403-404).**

486 There was little difference between the selection practices between NeT and NeS until late in the study. In the absence of extreme inbreeding along with extreme selection for the trait of interest, little reduction in genetic variation would be anticipated. **The NeT line is selected for the highest differentials to increase postweaning weight and RFI, which may contribute to reducing the selection pressure on weight in this line (L483-487).**

527 This is to be expected since there is a strong genetic correlation between weight and SC and little genetic correlation between weight and DFC. **The sentence was reformulated and references were added, since we did not analyze the correlations between these traits (L530-533).**

**Other text changes:**

**- All words "reclassified" have been replaced by "reranking".**
